# Synthesis and Antibacterial Evaluation of Novel 3-Substituted Ocotillol-Type Derivatives as Leads

**DOI:** 10.3390/molecules22040590

**Published:** 2017-04-07

**Authors:** Yi Bi, Xian-Xuan Liu, Heng-Yuan Zhang, Xiao Yang, Ze-Yun Liu, Jing Lu, Peter John Lewis, Chong-Zhi Wang, Jin-Yi Xu, Qing-Guo Meng, Cong Ma, Chun-Su Yuan

**Affiliations:** 1 School of Pharmacy, Key Laboratory of Molecular Pharmacology and Drug Evaluation (Yantai University), Ministry of Education, Collaborative Innovation Center of Advanced Drug Delivery System and Biotech Drugs in Universities of Shandong, Yantai University, Yantai 264005, China; beeyee_413@163.com (Y.B.); opendekevin@163.com (X.-X.L.); 18766565610@163.com (Z.-Y.L.); tainche@126.com (J.L.); 2 State Key Laboratory of Natural Medicines and Department of Medicinal Chemistry, China Pharmaceutical University, Nanjing 210009, China; panacea0928@163.com; 3 Department of Microbiology, The Chinese University of Hong Kong, Prince of Wales Hospital, Shatin, Hong Kong, China; xiaoyang@cuhk.edu.hk; 4 Biological Sciences, School of Environmental and Life Sciences, University of Newcastle, Callaghan, NSW 2308, Australia; peter.lewis@newcastle.edu.au; 5 Tang Center for Herbal Medicine Research, the University of Chicago, Chicago, IL 60637, USA; czwang@dacc.uchicago.edu (C.-Z.W.); cyuan@dacc.uchicago.edu (C.-S.Y.); 6 Department of Anesthesia and Critical Care, The University of Chicago, Chicago, IL 60637, USA; 7 Department of Applied Biology and Chemical Technology, and State Key Laboratory of Chirosciences, The Hong Kong Polytechnic University, Hung Hom, Kowloon, Hong Kong, China

**Keywords:** ocotillol, derivatives, synthesis, antibacterial activity, synergistic effect

## Abstract

Due to the rapidly growing bacterial antibiotic-resistance and the scarcity of novel agents in development, bacterial infection is still a global problem. Therefore, new types of antibacterial agents, which are effective both alone and in combination with traditional antibiotics, are urgently needed. In this paper, a series of antibacterial ocotillol-type C-24 epimers modified from natural 20(*S*)-protopanaxadiol were synthesized and evaluated for their antibacterial activity. According to the screening results of Gram-positive bacteria (*B. subtilis* 168 and MRSA USA300) and Gram-negative bacteria (*P. aer* PAO1 and *A. baum* ATCC19606) in vitro, the derivatives exhibited good antibacterial activity, particularly against Gram-positive bacteria with an minimum inhibitory concentrations (MIC) value of 2–16 µg/mL. The subsequent synergistic antibacterial assay showed that derivatives **5c** and **6c** enhanced the susceptibility of *B. subtilis* 168 and MRSA USA300 to chloramphenicol (CHL) and kanamycin (KAN) (FICI < 0.5). Our data showed that ocotillol-type derivatives with long-chain amino acid substituents at C-3 were good leads against antibiotic-resistant pathogens MRSA USA300, which could improve the ability of KAN and CHL to exhibit antibacterial activity at much lower concentrations with reduced toxicity.

## 1. Introduction

Therapeutic control of multidrug resistant bacteria has been emphasized in the area of global public health [1]. The generation of drug-resistant bacteria, such as MRSA USA300, has developed much faster than the development of new antimicrobial drugs. As the most common bacteria in blood-borne infection, MRSA USA 300 could cause lethal diseases, including severe sepsis, necrotizing pneumonia, and necrotizing fasciitis, all of which pose a serious threat to human health [2,3,4,5,6]. The first choice for the treatment of MRSA in clinic is vancomycin. However, the unreasonable use of vancomycin has further led to the production of vancomycin resistant strains [7]. Additionally, there are many side effects associated with long-term drug use. For example, chloramphenicol (CHL) can lead to aplastic anemia, granulocyte deficiency, double infection and gray baby syndrome [8,9]. Kanamycin (KAN) has many side effects, including ototoxicity, renal toxicity, hematopoietic system toxicity and neuromuscular blocking effects etc. [10,11]. Therefore, there is an urgent need to develop novel antibacterial agents to cure antibiotic-resistant bacteria infections, which can be used alone or in combination with traditional antibiotics to enhance their effectiveness and reduce their side effects.

Natural products have been the most effective source of leading compounds for the development of drugs, particularly anticancer and anti-infective agents [12]. Natural triterpenoids, including sterols, steroids and saponins, form a wide family of compounds, biosynthesized by cyclization reactions from 2,3-epoxysqualene [13]. These compounds have been used in traditional medicine and therefore have been studied for their anti-inflammatory, antimicrobial, anticancer and anti-HIV effects [14,15,16,17].

**Figure 1:**
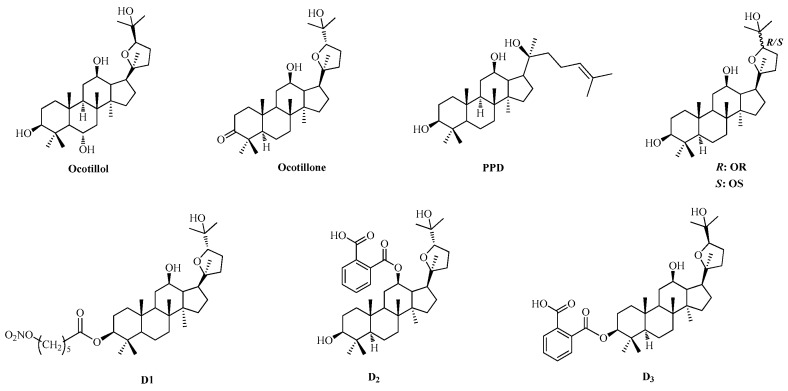
The structures of ocotillol and analogs.

Ocotillol (Figure 1), isolated from *Fouquieria splendens engelm*, bears a characteristic tetrahydrofuran ring at C-20 [18]. Studies have shown that ocotillone (Figure 1) isolated from the stem bark of *ailanthus altisshima* has potent antibacterial activities against *P. aeruginosa* and *S. typhimurium*, without hemolytic activity [19]. In our previous studies, ocotillol-type epimers **OR** and **OS** were synthesized from 20(*S*)-protopanaxadiol (PPD) (Figure 1) [20]. Many ocotillol-type analogs, such as **OS**, **D1**, **D2**, **D3** (Figure 1), showed antibacterial activity against Gram-positive bacteria and demonstrated outstanding synergistic effects with CHL and KAN. For example, the minimum inhibitory concentrations (MIC) of KAN combining with **D1** against MRSA USA300 was reduced from 0.25 µg/mL to 0.0625 µg/mL [21,22,23]. Thus, ocotillol analogs with good synergistic activity against MRSA USA300 hold the potential to become leads of novel antibiotic-resistant agents with unique mechanisms. The synthesis of new ocotillol-type derivatives, followed by antibacterial evaluations, are described in this study.

## 2. Results and Discussion

### 2.1. Synthesis of Ocotillol-Type Derivatives

**Scheme 1:**
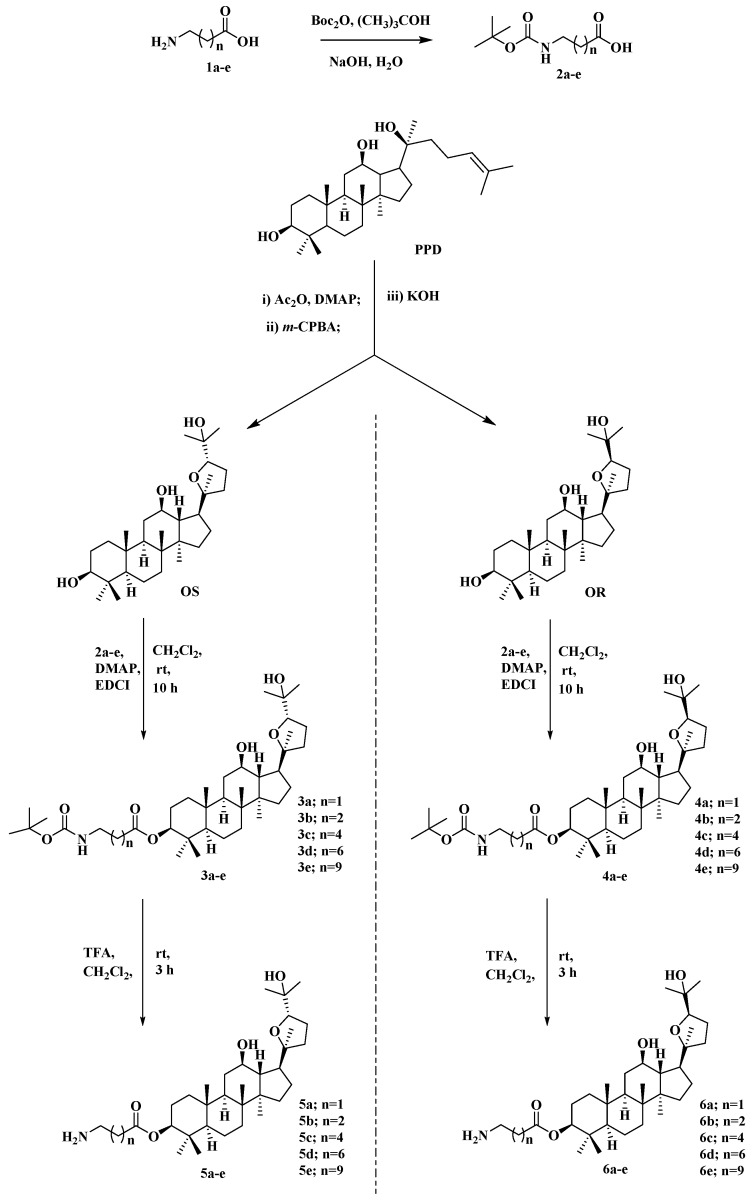
Synthesis of ocotillol-type derivatives **5a**–**e** and **6a**–**e**.

As previously described [20], in the synthesis of epimers **OS** and **OR**, PPD was chosen as the starting material. Firstly, PPD was protected with acetic anhydride under the catalysis of DMAP, then oxidized by *m*-CPBA, and finally deprotected by potassium hydroxide to obtain **OS** and **OR** with the desired yields. **OS** was reacted with a series of carboxylic acids to give intermediates **3a**–**e**, and finally target compounds **5a**–**e** were obtained by deprotection using trifluoroacetic acid. Referring to the synthesis of **5a**–**e**, **6a**–**e** were gained from **OR** as the starting material (Scheme 1).

### 2.2. Antibacterial Activity

**Figure 2:**
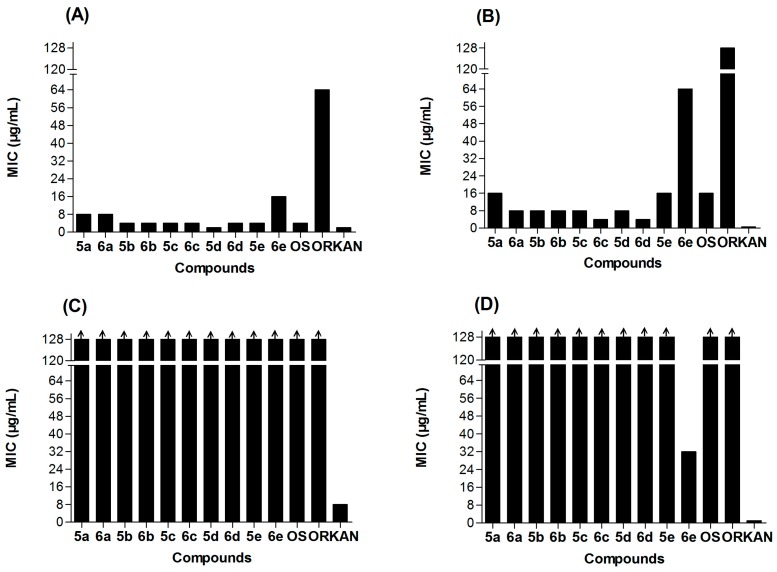
In vitro antibacterial activity of ocotillol-type derivatives against (**A**) *B. subtilis* 168; (**B**) MRSA USA300; (**C**) *P. aer* PAO1 and (**D**) *A. baum* ATCC19606. “↑”: MIC > 128 µg/mL.

Initial MIC screening results are shown in Figure 2. The results showed that most of the ocotillol-type derivatives had moderate to good inhibitory activity against Gram-positive bacteria with MIC values of about 2–32 µg/mL, but had no effect on Gram-negative bacteria. The data illustrated that, with unprotected primary amine groups, all target compounds showed good inhibitory activity against *B. subtilis* 168 with MIC values of about 2–16 µg/mL, and against MRSA USA300 with MIC values of about 4–16 µg/mL, except **6e**. The study found that, among the synthesized compounds, **5d** was the most active compound against *B. subtilis* 168, with a MIC value of 2 µg/mL, while **6c** and **6d** showed the most potent activity against MRSA USA300, with a MIC value of 4 µg/mL. Among these derivatives, compound **6e** had the weakest activity against Gram-positive bacteria but only a moderate inhibitory activity against *A. Baum* ATCC19606, with a MIC value of 32 µg/mL. Additionally, a similar inhibitory activity against Gram-positive bacteria was observed between epimers **5a**–**d** and **6a**–**d**, but a dramatically dissimilar one was present between **5e** and **6e**, which suggested that the spatial configuration of the isopropanol group at C-24 and the length of the C-3 side chain affected both its conformation and bioactivity.

**Figure 3:**
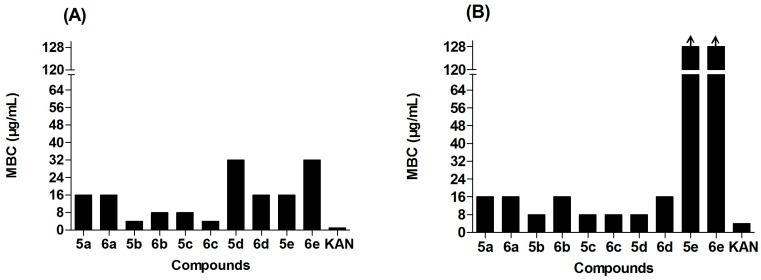
In vitro bactericidal activity of ocotillol-type derivatives against (**A**) *B. subtilis* 168 and (**B**) MRSA USA300. “↑”: MIC > 128 µg/mL.

As shown in Figure 3, the minimum bactericidal concentration (MBC) was measured for compounds **5a**–**e** and **6a**–**e** against *B. subtilis* 168 and MRSA USA300. The derivatives with bactericidal activities in these strains warrant further studies as anti-bacterial leads. The results showed that compounds **5a**–**e** and **6a**–**e** possess good bactericidal activity against *B. subtilis* 168 with MBC values of 4–32 µg/mL, and compounds **5a**–**d** and **6a**–**d** possess good bacterial activity against MRSA USA300 with MBC values of 8–16 µg/mL. Compounds **5b**, **5c** and **6c** could be candidates since they possess the most potent activities against the community-acquired meticillin-resistant strain MRSA USA300 and laboratory strain *B. subtilis* 168.

### 2.3. Synergistic Antibacterial Activity

**Figure 4:**
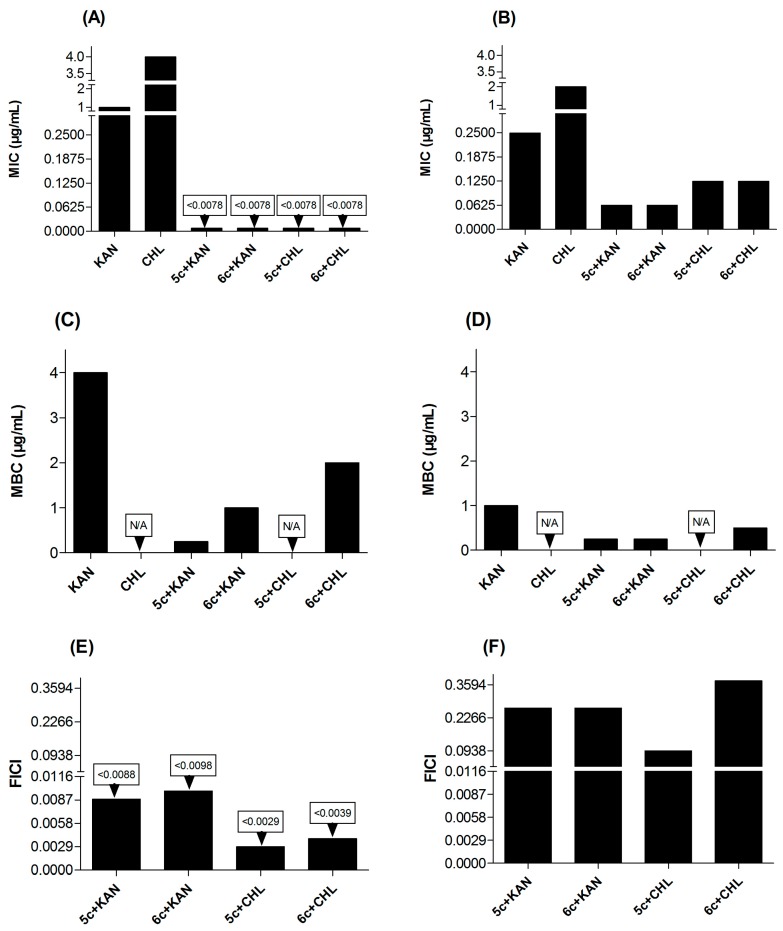
Synergistic effect of different antibiotics with compounds **5c** and **6c** against MRSA USA300 and *B. subtilis* 168. (**A**) MIC of different compounds against MRSA USA300 and (**B**) *B. subtilis* 168; (**C**) MBC of different compounds against MRSA USA300 and (**D**) *B. subtilis* 168; (**E**) FICI of different compounds against MRSA USA300 and (**F**) *B. subtilis* 168.

According to Figure 4, the MIC of kanamycin (KAN) and chloramphenicol (CHL) against MRSA USA300 and *B. subtilis* 168 significantly decreased when combining compounds **5c** and **6c** at half-MIC, and the synergistic effect was significant (FICI < 0.5, According to the literature [24]: FICI = FIC_A_ + FIC_B_ = (MIC_A+B_/MIC_A_) + (MIC_A+B_/MIC_B_). When FICI value was less than or equal to 0.5, it has a synergistic effect). Compounds **5c** and **6c** could decrease the MICs of KAN or CHL against MRSA USA300, and the MICs were all less than 0.0078 µg/mL (FICI < 0.0088, 0.0098, 0.0029, 0.0039). Synergistic activity against *B. subtilis* 168 was also observed during the combination of **5c** and **6c** with KAN or CHL (FICI = 0.2656, 0.2656, 0.0938, 0.3750). In contrast, when **5c** (or **6c**) was combined with KAN, the bactericidal activity of KAN against MRSA USA300 was significantly enhanced from 4 µg/mL to 0.25 (or 1) µg/mL. For *B. subtilis* 168, potent bactericidal effects were also observed during the combination of KAN with **5c** and **6c**. Surprisingly, CHL alone was bacteriostatic, but possessed bactericidal effect when combined with **6c**, with MBC value of 2 µg/mL against MRSA USA 300 and 0.5 µg/mL against *B. subtilis* 168.

### 2.4. The Structure-Activity Relationships (SARs) of Ocotillol-Type Derivatives

Based on the previous and present data of ocotillol-type derivatives, preliminary SARs can be summarized. The (24*S*)-configuration is preferred for pharmacological activity of compounds without substitution at 3-OH; substitution at 3-OH may cause changes of molecular conformation resulting in bioactive (24*R*)-compounds [21,25]; hydrogen donors at C-3 positions are the effective group and enhance the antibacterial activity against Gram-positive bacteria [21,23,25]; the length of 4–8 carbon atoms at the C-3 side chain is preferred if there is -NH_2_ on the side chain; however, the substitution of 11 carbon atoms at the C-3 side chain resulted in mild activity against Gram-negative bacteria, in particular compound **6e** with (24*R*)-configuration showed specific activity against Gram-negative bacteria.

## 3. Materials and Methods

### 3.1. Chemical Reagents and Instruments

The majority of chemicals and reagents used in the experiment was analytically pure and was purified and dried with standard methods when necessary. ^1^H-NMR and ^13^C-NMR spectra were measured using a Bruker AV-300 spectrometer in the specific solvents (Tetramethyl silane (TMS) as internal standard): the chemical shifts are expressed with *δ* values (ppm) and the coupling constants (*J*) with Hz. High-resolution mass spectra were measured using an Agilent QTOF 6520.

### 3.2. General Procedure for the Synthesis of (20S,24S)-Epoxy-dammarane-3β,12β,25-triol (***OS***) and (20S,24R)–Epoxy-dammarane-3β,12β,25-triol (***OR***)

Compounds **OR** and **OS** were prepared from PPD according to the published procedures [18,21].

### 3.3. General Procedure for the Synthesis of Compounds ***5a**–**e*** and ***6a**–**e***

To a solution of sodium hydroxide (800 mg, 19.5 mmol) and di-tert-butyl dicarbonate (4.3 g, 19.5 mmol) in tertiary butyl alcohol (11 mL) and H_2_O (9 mL) the corresponding amino acids **1a**–**e** (19.5 mmol) were added. After the mixture was stirred at room temperature for 16 h, diluted with 10% HCl (100 mL), and extracted with ethyl acetate, and the organic layer was washed with water and brine successively, dried over anhydrous sodium sulfate, filtered and concentrated to give **2a**–**e**.

To a solution of **2a**–**e** (1.5 eq.), dry dichloromethane EDCI (1.0 eq.) and DMAP (1.0 eq.) were added, and the mixture was stirred at room temperature for 1 h. Then, **OS** (or **OR**) (1.0 eq.) was added. After stirring for 12 h, the mixture was removed in vacuo and diluted by ethyl acetate, washed with water and brine, dried over anhydrous sodium sulfate, filtered, concentrated, and purified by column chromatography over silica gel (8:1–6:1 petroleum ether: ethyl acetate) to give **3a**–**e** (or **4a**–**e**). 

To a solution of intermediates **3a**–**e** (or **4a**–**e**) (1 eq.), trifluoroacetic acid (2 eq.) was added under an ice bath. After stirring at room temperature for 3 h, the mixture was removed in vacuo and purified by column chromatography over silica gel (30:1–10:1 dichloromethane: methanol) to give **5a**–**e** (or **6a**–**e**).

*(20S,24S)-Epoxy-3β-O-(3-aminopropionyl)-dammarane-12β,25-diol* (**5a**). Yellow oily matter, 72% yield. ^1^H-NMR (300 MHz, CDCl_3_) δ 4.51 (m, 1H, -OCH-), 3.87 (dd, *J* = 10.3Hz, 5.1Hz, 1H, -OCH-), 3.53 (td, *J* = 10.2 Hz, 4.6 Hz, 1H, -OCH-), 3.05 (t, 2H, -NCH_2_-), 2.56 (t, *J* = 6.3 Hz, 2H, -CH_2_CO-), 1.27 (s, 3H, CH_3_), 1.23 (s, 3H, CH_3_), 1.11 (s, 3H, CH_3_), 1.01 (s, 3H, CH_3_), 0.91 (s, 6H, CH_3_), 0.85 (s, 6H, CH_3_); ^13^C-NMR (75 MHz, CDCl_3_) δ 171.9, 87.4, 87.1, 81.4, 70.5, 70.2, 56.0, 52.1, 50.1, 48.9, 48.8, 39.8, 38.6, 37.9, 37.1, 34.7, 32.2, 31.7, 29.3, 28.9, 28.5, 28.0, 27.8, 27.4, 26.1, 25.2, 24.1, 23.7, 18.2, 17.8, 16.4, 16.3, 15.4. ESI-MS: *m*/*z* [M + H]^+^: 548.4. 

*(20S,24S)-Epoxy-3β-O-(4-aminobutyryl)-dammarane-12β,25-diol* (**5b**). Yellow oily matter, 69% yield. ^1^H-NMR (300 MHz, CDCl_3_) δ 4.47 (m, 1H, -OCH-), 3.87 (dd, *J* = 10.4 Hz, 5.0 Hz, 1H, -OCH-), 3.53 (td, *J* = 10.1 Hz, 4.4 Hz, 1H, -OCH-), 3.07 (t, *J* = 7.2 Hz, 2H, -NCH_2_-), 2.48 (t, *J* = 6.9 Hz, 2H, -CH_2_CO-), 1.27 (s, 3H, CH_3_), 1.22 (s, 3H, CH_3_), 1.11 (s, 3H, CH_3_), 1.01 (s, 3H, CH_3_), 0.92 (s, 3H, CH_3_), 0.91 (s, 3H, CH_3_), 0.85 (s, 3H, CH_3_), 0.83 (s, 3H, CH_3_); ^13^C-NMR (75 MHz, CDCl_3_) δ 172.7, 87.4, 87.1, 81.5, 70.5, 70.2, 56.0, 52.1, 50.1, 48.9, 48.8, 39.8, 39.3, 38.6, 37.9, 37.1, 34.7, 32.2, 31.7, 31.5, 29.3, 28.9, 28.5, 28.1, 27.8, 25.2, 24.1, 23.7, 22.8, 18.2, 17.8, 16.5, 16.3, 15.5. ESI-MS: *m*/*z* [M + H]^+^: 562.4.

*(20S,24S)-Epoxy-3β-O-(4-aminohexanoyl)-dammarane-12β,25-diol* (**5c**). Yellow oily matter, 68% yield. ^1^H-NMR (300 MHz, CDCl_3_) δ 4.47 (m, 1H, -OCH-), 3.87 (dd, *J* = 10.4 Hz, 5.0 Hz, 1H, -OCH-), 3.52 (td, *J* = 10.1 Hz, 4.4 Hz, 1H, -OCH-), 2.99 (t, *J* = 7.5 Hz, 2H, -NCH_2_-), 2.32 (t, *J* = 7.1 Hz, 2H, -CH_2_CO-), 1.27 (s, 3H, CH_3_), 1.22 (s, 3H, CH_3_), 1.12 (s, 3H, CH_3_), 1.01 (s, 3H, CH_3_), 0.92 (s, 3H, CH_3_), 0.91 (s, 3H, CH_3_), 0.85 (s, 3H, CH_3_), 0.83 (s, 3H, CH_3_); ^13^C-NMR (75 MHz, CDCl_3_) δ 173.4, 87.4, 87.1, 80.8, 70.5, 70.2, 56.0, 52.1, 50.1, 48.9, 48.8, 39.8, 38.6, 37.9, 37.1, 34.7, 34.4, 32.2, 31.7, 31.0, 28.9, 28.5, 28.0, 27.8, 27.7, 27.1, 26.0, 25.2, 24.4, 24.1, 23.7, 18.2, 17.8, 16.5, 16.3, 15.5. ESI-MS: *m*/*z* [M + H]^+^: 590.4.

*(20S,24S)-Epoxy-3β-O-(8-aminooctanoyl)-dammarane-12β,25-diol* (**5d**). Yellow oily matter, 66% yield. ^1^H-NMR (300 MHz, CDCl_3_) *δ* 4.47 (m, 1H, -OCH-), 3.88 (dd, *J* = 8.3 Hz, 6.9 Hz, 1H, -OCH-), 3.53 (td, *J* = 10.3 Hz, 4.4 Hz, 1H, -OCH-), 2.96 (t, *J* = 7.2 Hz, 2H, -NCH_2_-), 2.29 (t, *J* = 7.4 Hz, 2H, -CH_2_CO-), 1.82–2.09 (m, 6H, CH_2_), 1.27 (s, 3H, CH_3_), 1.26 (s, 3H, CH_3_), 1.10 (s, 3H, CH_3_), 1.01 (s, 3H, CH_3_), 0.91 (s, 6H, CH_3_), 0.86 (s, 3H, CH_3_), 0.84 (s, 3H, CH_3_); ^13^C-NMR (75 MHz, CDCl_3_) δ 173.5, 87.4, 87.1, 80.6, 70.5, 70.2, 56.0, 52.1, 50.2, 48.9, 48.8, 39.9, 39.8, 38.6, 37.9, 37.1, 34.7, 32.2, 31.7, 31.2, 28.9, 28.6, 28.5, 28.0, 27.9, 27.5, 27.4, 26.3, 26.2, 25.2, 24.9, 24.1, 23.7, 18.2, 17.8, 16.5, 16.3, 15.5. ESI-MS: *m*/*z* [M + H]^+^: 618.5.

*(20S,24S)-Epoxy-3β-O-(11-undecanoyl)-dammarane-12β,25-diol* (**5e**). Yellow oily matter, 61% yield. ^1^H-NMR (300 MHz, CDCl_3_) δ 4.48 (dd, *J* = 10.1 Hz, 5.9 Hz, 1H, -OCH-), 3.88 (dd, *J* = 10.4 Hz, 5.0 Hz, 1H, -OCH-), 3.51 (td, *J* = 10.3 Hz, 5.6 Hz, 1H, -OCH-), 2.96 (t, *J* = 7.5 Hz, 2H, -NCH_2_-), 2.29 (t, *J* = 7.4 Hz, 2H, -CH_2_CO-), 1.78-2.06 (m, 6H, CH_2_), 1.27 (s, 3H, CH_3_), 1.26 (s, 3H, CH_3_), 1.10 (s, 3H, CH_3_), 1.01 (s, 3H, CH_3_), 0.91 (s, 6H, CH_3_), 0.86 (s, 3H, CH_3_), 0.85 (s, 3H, CH_3_); ^13^C-NMR (75 MHz, CDCl_3_) δ 173.6, 87.4, 87.1, 80.4, 70.5, 70.2, 56.0, 52.1, 50.1, 48.9, 48.8, 48.0, 40.0, 39.8, 38.6, 37.9, 37.1, 34.8, 32.2, 31.7, 31.4, 29.3, 29.2, 29.1, 29.0, 28.9, 28.5, 28.0, 27.8, 27.6, 26.5, 26.2, 25.2, 25.1, 24.1, 23.7, 18.2, 17.8, 16.5, 16.3, 15.4. ESI-MS: *m*/*z* [M + H]^+^: 660.5.

*(20S,24R)-Epoxy-3β-O-(3-aminopropionyl)-dammarane-12β,25-diol* (**6a**). Yellow oily matter, 79% yield. ^1^H-NMR (300 MHz, CDCl_3_) δ 4.51 (m, 1H, -OCH-), 3.84 (dd, *J* = 8.3 Hz, 6.9 Hz, 1H, -OCH-), 3.51 (td, *J* = 10.4 Hz, 4.4 Hz, 1H, -OCH-), 3.28 (t, 2H, -NCH_2_-), 2.83 (t, 2H, -CH_2_CO-), 1.27 (s, 3H, CH_3_), 1.26 (s, 3H, CH_3_), 1.10 (s, 3H, CH_3_), 0.98 (s, 3H, CH_3_), 0.90 (s, 3H, CH_3_), 0.87 (s, 3H, CH_3_), 0.84 (s, 6H, CH_3_); ^13^C-NMR (75 MHz, CDCl_3_) δ 171.1, 86.5, 85.3, 82.2, 70.9, 70.1, 56.0, 52.0, 50.4, 49.3, 48.0, 39.8, 38.6, 37.9, 37.0, 35.8, 32.6, 31.7, 31.3, 31.1, 28.6, 28.1, 27.9, 27.5, 26.1, 25.0, 24.0, 23.6, 18.2, 17.5, 16.4, 16.3, 15.4. ESI-MS: *m*/*z* [M + H]^+^: 548.4.

*(20S,24R)-Epoxy-3β-O-(4-aminobutyryl)-dammarane-12β,25-diol* (**6b**). Yellow oily matter, 72% yield. ^1^H-NMR (300 MHz, CDCl_3_) δ 4.47 (m, 1H, -OCH-), 3.85 (dd, *J* = 10.4 Hz, 5.0 Hz, 1H, -OCH-), 3.52 (td, *J* = 10.1 Hz, 4.4 Hz, 1H, -OCH-), 3.05 (t, *J* = 7.2 Hz, 2H, -NCH_2_-), 2.47 (t, *J* = 6.9 Hz, 2H, -CH_2_CO-), 1.27 (s, 3H, CH_3_), 1.23 (s, 3H, CH_3_), 1.11 (s, 3H, CH_3_), 1.01 (s, 3H, CH_3_), 0.92 (s, 3H, CH_3_), 0.91 (s, 3H, CH_3_), 0.85 (s, 3H, CH_3_), 0.83 (s, 3H, CH_3_). ^13^C-NMR (75 MHz, CDCl_3_) δ 172.7, 86.5, 85.3, 81.1, 70.5, 70.2, 56.0, 52.1, 50.1, 48.9, 48.8, 39.8, 39.3, 38.5, 37.9, 37.1, 34.6, 32.2, 31.7, 31.5, 29.3, 28.8, 28.5, 28.1, 27.9, 25.2, 24.1, 23.6, 22.8, 18.2, 17.9, 16.5, 16.3, 15.5. ESI-MS: *m*/*z* [M + H]^+^: 562.4.

*(20S,24R)-Epoxy-3β-O-(4-aminohexanoyl)-dammarane-12β,25-diol* (**6c**). Yellow oily matter, 70% yield. ^1^H-NMR (300 MHz, CDCl_3_) *δ* 4.46 (m, 1H, -OCH-), 3.85 (dd, *J* = 8.6 Hz, 6.8 Hz, 1H, -OCH-), 3.52 (td, *J* = 10.2 Hz, 4.2 Hz, 1H, -OCH-), 2.97 (t, *J* = 7.6 Hz, 2H, -NCH_2_-), 2.32 (t, *J* = 7.3 Hz, 2H, -CH_2_CO-), 1.89–2.11 (m, 2H, CH_2_), 1.27 (s, 3H, CH_3_), 1.27 (s, 3H, CH_3_), 1.10 (s, 3H, CH_3_), 0.98 (s, 3H, CH_3_), 0.90 (s, 3H, CH_3_), 0.88 (s, 3H, CH_3_), 0.84 (s, 3H, CH_3_), 0.83 (s, 3H, CH_3_); ^13^C-NMR (75 MHz, CDCl_3_) δ 173.4, 86.5, 85.3, 80.8, 70.5, 70.2, 56.0, 52.1, 50.1, 48.9, 48.8, 39.8, 38.6, 37.9, 37.1, 34.7, 34.3, 32.5, 31.3, 31.1, 29.1, 28.6, 28.0, 27.8, 27.5, 27.0, 25.8, 25.0, 24.3, 24.1, 23.7, 18.1, 17.8, 16.4, 16.3, 15.4. ESI-MS: *m*/*z* [M + H]^+^: 590.4.

*(20S,24R)-Epoxy-3β-O-(8-aminooctanoyl)-dammarane-12β,25-diol* (**6d**). Yellow oily matter, 70% yield. ^1^H-NMR (300 MHz, CDCl_3_) δ 4.46 (m, 1H, -OCH-), 3.84 (dd, *J* = 8.3 Hz, 6.9 Hz, 1H, -OCH-), 3.52 (td, *J* = 10.3 Hz, 4.4 Hz, 1H, -OCH-), 2.95 (t, *J* = 7.2 Hz, 2H, -NCH_2_-), 2.28 (t, *J* = 7.4 Hz, 2H, -CH_2_CO-), 1.90–2.10 (m, 2H, CH_2_), 1.82-1.90 (m, 2H, CH_2_), 1.27 (s, 3H, CH_3_), 1.26 (s, 3H, CH_3_), 1.10 (s, 3H, CH_3_), 0.98 (s, 3H, CH_3_), 0.90 (s, 3H, CH_3_), 0.88 (s, 3H, CH_3_), 0.84 (s, 3H, CH_3_), 0.83 (s, 3H, CH_3_); ^13^C-NMR (75 MHz, CDCl_3_) δ 173.6, 86.5, 85.3, 80.6, 70.9, 70.1, 56.1, 52.0, 50.4, 49.3, 48.0, 39.9, 39.8, 38.6, 37.9, 37.1, 34.8, 32.6, 31.3, 31.1, 28.8, 28.6, 28.5, 28.0, 27.9, 27.5, 27.4, 26.2, 26.1, 25.0, 24.9, 24.1, 23.7, 18.1, 17.7, 16.5, 16.4, 15.4. ESI-MS: *m*/*z* [M + H]^+^: 618.5.

*(20S,24R)-Epoxy-3β-O-(11-undecanoyl)-dammarane-12β,25-diol* (**6e**). Yellow oily matter, 67% yield. ^1^H-NMR (300 MHz, CDCl_3_) *δ* 4.47 (dd, *J* = 9.9 Hz, 5.6 Hz, 1H, -OCH-), 3.85 (dd, *J* = 8.6 Hz, 6.8 Hz, 1H, -OCH-), 3.51 (td, *J* = 10.2 Hz, 6.1 Hz, 1H, -OCH-), 2.96 (t, *J* = 7.3 Hz, 2H, -NCH_2_-), 2.29 (t, *J* = 7.4 Hz, 2H, -CH_2_CO-), 1.80–2.08 (m, 6H, CH_2_), 1.27 (s, 3H, CH_3_), 1.26 (s, 3H, CH_3_), 1.10 (s, 3H, CH_3_), 0.98 (s, 3H, CH_3_), 0.90 (s, 3H, CH_3_), 0.88 (s, 3H, CH_3_), 0.85 (s, 3H, CH_3_), 0.84 (s, 3H, CH_3_); ^13^C-NMR (75 MHz, CDCl_3_) δ 173.6, 86.5, 85.3, 80.4, 70.9, 70.1, 56.1, 52.0, 50.4, 49.3, 48.8, 48.0, 40.0, 39.8, 38.6, 37.9, 37.1, 34.8, 32.6, 31.3, 31.1, 29.3, 29.2, 29.1, 29.0, 28.6, 28.5, 28.0, 27.9, 27.5, 26.5, 26.1, 25.1, 25.0, 24.0, 23.7, 18.1, 17.6, 16.5, 16.4, 15.4. ESI-MS: *m*/*z* [M + H]^+^: 660.5.

### 3.4. Pharmacology

The antibacterial and synergistic activity experiment was carried out as described previously [23,26]. The antibacterial activity was screened in vitro, and the minimum inhibitory concentrations (MIC) were determined against Gram-positive bacteria (*B. subtilis* 168 and MRSA USA300) and Gram-negative bacteria (*P. aeruginosa* PAO1 and *A. baumannii* ATCC19606). These pathogens have been commonly used to screen antibacterial compounds. Compounds with good inhibitory activity were selected to determine their bactericidal activity. The minimum bactericidal concentration (MBC) was determined against *B. subtilis* 168 and MRSA USA300 using a standard LB medium dilution technique. *B. subtilis* 168 was the tool strain used to research the antibacterial mechanism. MRSA USA300 was the drug-resistant strain used to evaluate anti-drug-resistant candidates [4,22]. Kanamycin was used as a positive control. 

## 4. Conclusions

In summary, ten novel 3-substituted ocotillol-type derivatives from natural PPD were synthesized and evaluated for antimicrobial activity. According to the results of antibacterial tests in vitro, derivatives with a primary amine at C-3 were found, and these compounds possessed good antibacterial activity against Gram-positive bacteria, such as *B. subtilis* 168 and MRSA USA300. The synergistic antibacterial assay showed that **5c** and **6c** could enhance the susceptibility of *B. sub* 168 and MRS USA300 to KAN and CHL (FICI < 0.5).

These results showed that ocotillol-type derivatives **5c** and **6c** are promising leads to develop novel antibacterial agents against many community-associated and health care-associated infections caused by MRSA USA300. Further studies will be conducted to determine the bactericidal functional mechanisms of these compounds.
